# Serial brain natriuretic peptide strongly predicts in-hospital mortality in patients with acute myocardial infarction

**DOI:** 10.1186/cc12622

**Published:** 2013-06-19

**Authors:** AE Pereira Pesaro, M Katz, C Pereira, AG Correa, AN Fava, M Franken, ACB Nunes, LM Forlenza, F Tarasoutchi, MR Makdisse

**Affiliations:** 1 Hospital Israelita Albert Einstein, Morumbi, São Paulo, SP, Brazil

## Introduction

Brain natriuretic peptide (BNP) can be useful in risk stratification of patients with acute myocardial infarction (AMI). However, the value of serial in-hospital BNP assessment to predict mortality in this setting was poorly investigated before. Thus, the aim of this study was to evaluate the usefulness of serial BNP measurements to predict mortality in patients with AMI.

## Methods

Patients with AMI (*n = *2,198) were consecutively enrolled between 2004 and 2012 in a prospective single-center registry. A subgroup analysis was performed in the patients submitted to serial BNP measurements as indicated for clinical purposes. The BNP variation was calculated based on the change between the first BNP collected (baseline) and the highest subsequent in-hospital BNP measurement. Baseline characteristics and the BNP variation were compared between patients who survived or died during the in-hospital period. Categorical variables were compared by Pearson's chi-square test. Continuous variables were compared using the Student *t *test or Mann-Whitney test. The logistic regression model was used to test the association between BNP increase (categorized by an increase ≥200 pg/ml) and mortality, adjusted for age, gender, troponin levels, Killip classification, left ventricular ejection fraction (LVEF) and ST elevation AMI. *P *<0.05 was considered statistically significant.

## Results

Serial BNP levels were determined in 280 patients (59% men, 78 ± 12 years, 33% ST elevation). The BNP increase >200 pg/ml was detected in 114 (41%) patients. All baseline clinical parameters (gender, age, diabetes, hypertension, Killip classification, LVEF and ST elevation) were similar between patients with or without BNP increase. Mortality was higher in patients with BNP increase (25% vs. 12%; *P *= 0.006). In the adjusted logistic regression model, only age (OR = 1.04; 95% CI = 1.002 to 1.08; *P *= 0.04) and BNP increase >200 pg/ml (OR = 3.9; 95% CI = 1.83 to 8.20; *P *<0.001) were independent predictors of in-hospital mortality.

## Conclusion

This study demonstrated that an in-hospital BNP increase >200 pg/ml is strongly and independently related to in-hospital mortality in patients with AMI. Thus, serial BNP testing may be useful to detect high-risk AMI patients.

